# Development of a scale to evaluate medical professional identity formation

**DOI:** 10.1186/s12909-019-1499-9

**Published:** 2019-02-28

**Authors:** Masami Tagawa

**Affiliations:** Center for Innovation in Medical and Dental Education, Graduate School of Medical and Dental Sciences, Kagoshima University, 8-35-1 Sakuragaoka, Kagoshima, 890-8544 Japan

**Keywords:** Professional identity formation, Evaluation, Medical trainees, Socialization, Role

## Abstract

**Background:**

Medical educators now focus on professional identity formation (PIF), which is a process of psychological development and socialization in the community of practice. This study aimed to develop an instrument to evaluate PIF that can be applied to a large group of medical trainees.

**Methods:**

A self-administered questionnaire was created with items on priorities, behavior standards, attitudes, and emotional control of well-developed physicians, in addition to items on their background and experience in playing the role of a physician. The participants were divided into four respondent groups: 4th- and 6th-year medical students and 2nd-year residents at Kagoshima University, and experienced medical doctors (instructors).

**Results:**

Using factor analysis of data from 318 respondents and respondent group comparison, a developing scale (DS) with 15 items was created.

The DS has a five-factor structure and evaluates self-control as a professional (factor 1), awareness of being a medical doctor (factor 2), reflection as a medical doctor (factor 3), execution of social responsibility (factor 4), and external and internal self-harmonization (factor 5). The mean DS score of the instructors was significantly higher than that of the residents (*p* < 0.01), the mean score of residents and instructors was significantly higher than that of students (*p* < 0.01), and the mean score of instructors was significantly higher than that of all other respondents (*p* < 0.01). Respondent group, but not gender, was a significant variable of the DS. The DS and scores of factors 2 and 4 correlated with 6th-year medical students’ experience in playing the role of a physician during clinical training, and scores of factors 3 and 4 correlated with 2nd-year residents’ experience in playing the role of a physician. There was no significant difference between the mean DS score of 4th- and 6th-year medical students, which might due to less clinical experience among 6th-year medical students or a limitation of the scale to evaluate pre-clinical medical students.

**Conclusions:**

The DS could be a useful indicator of medical trainees’ personal and professional development and socialization. Experience in playing the role of a physician might facilitate medical trainees’ PIF.

## Background

In recent years, medical educators have focused on the acquisition of professional identity, or professional identity formation (PIF), as a medical doctor [1–5] as the ultimate goal of medical education [6]. Most medical educators try to teach and assess medical trainees’ measurable behaviors under the framework of competency-based medical education [7]. Jarvis-Selinger described identity formation as an adaptive, developmental process that happens simultaneously at the individual and collective levels and involves psychological development and socialization of the person into appropriate roles and forms of participation in the community’s work [7]. Socialization in the community of practice and acceptance of professional values [8, 9] could be indispensable core features of PIF in medical education. However, medical students and residents face various experiences and difficulties in the process of PIF [4, 10–16], and it is important for medical educators to understand the PIF of learners in order to help them develop as medical professionals.

Previous research on medical students’ and residents’ values and attitudes used qualitative analysis of reflective writing and interviews. If medical educators intend to follow-up on an individual’s or group’s PIF, compare different groups, or evaluate medical educational programs from the standpoint of PIF, a quantitative assessment tool would be preferable and would broaden research possibilities.

A previous study indicated that Kegan’s human developmental model [17] could apply to medical professions and manifestation in a medical context was discussed [4, 7, 15]. Kegan’s model, which was based on Piaget [18] and other developmental theories [19, 20], indicates a framework for the longitudinal psychological development of the self into a moral and meaning-making entity. Kegan proposed six stages from childhood stage 0 to adult life stage 5, and this life-span development is the process of PIF from self-centered identity to a moral identity characterized by the expectation of a profession [1]. Previous research suggests that medical students and young trainees could be between Kegan’s stages 2 to 4. People at stage 4 understand relationships in terms of different values and expectations, become self-reflective, incorporate external professional values as internal values, and demonstrate reason in full control over emotions. Not all people reach stage 5, and those at stage 5 do not perceive themselves as having a single identity and are open to other influences. An evaluation scale could be developed using items on well-developed (stage 4 and higher) professional attributes as indicators.

The purpose of this study was to develop an instrument to evaluate the degree of maturation, socialization, and acceptance of professional values, which comprise the core features of PIF, and that can be applied to a large group of medical trainees.

## Methods

Assessing whether an individual has the core features of PIF requires the evaluation of his/her consistent demonstration of the attitude, values, and behaviors expected of one who has come to think, act, and feel like a physician [21]. The process of developing a scale to evaluate the core features of PIF is based on the following three principles:

1) as demonstrated in a previous cross-sectional qualitative study on military PIF [22], medical trainees’ attitudes and values might develop into those of advanced professionals as their level of education and clinical experience increase, even though trainees in the same program are expected to be at various stages of the PIF process.

2) Respondents’ answers to items on a scale asking about their habitual behaviors, experiences, and recognition and priorities in daily life and in specific situations reflect their conscious and unconscious attitudes and values.

3) One scale that consists of items that estimate the degree of maturation and socialization could evaluate medical trainees’ PIF.

### Attitudes and values to be measured and initial item development

One scale to assess the overall degree of PIF was created, according to descriptions of medical trainees’ personal characteristics and behaviors or attitudes manifested in a professional context based on previous studies [7, 15]. This scale was named the developing scale (DS) because it quantifies the state of individual maturation and professional development.

To develop the DS, items describing emotional control in several situations, recognition of professional role, internalization of external values and social requirements, daily reflection and self-evaluation behaviors which would be expected of medical doctors at higher stages of professional development were created. In addition, items evaluating preferences regarding social inclusion, which is typically seen between Kegan’s stage 3 and 5, and items describing stage transition were also included. Since lack of control of emotions and prioritizing one’s own needs and interests (stage 2-specific attributes) should not be characteristics of well-developed professionals, reverse scoring for such candidate items was applied for the DS. Initially, 51 items were listed, and then 31 generally applicable items covering various perspectives of well-developed professional attributes manifested in professional contexts and daily life, consisting of emotional control (3 items), recognition and persuasion of professional role (4 items), internalization of external values and social requirements (8 items), daily reflection and evaluation (5 items), social inclusion (8 items), and stage transition (3 items), were extracted and rewritten for the DS.

A self-administered anonymous questionnaire was created with these items. Each item was scored on a 7-point Likert scale that ranged from 1 (completely inapplicable) to 7 (greatly applicable), and 4 was neutral. Fifteen of the 31 items were reversely coded for data analysis.

The questionnaire also asked about demographic characteristics (gender, age), as well as work experience and position for instructors. Four items for 6th-year medical students and residents asked about experience in playing the role of a physician.

### Data collection

From July 2016 to March 2018, the printed questionnaire was distributed by hand to 4th-year medical students about to start their clinical clerkship courses, to 6th-year medical students who finished 1.5 years of all clinical clerkship courses, and to residents in the last month of the 2-year residency program at Kagoshima University. The questionnaire was also distributed by mail to experienced medical doctors working in community hospitals or private clinics in Kagoshima Prefecture who engaged in undergraduate medical education as senior instructors in January 2017. This study was approved by the Graduate School of Medical and Dental Sciences, Kagoshima University (No. 629 July 15, 2016, No. 666 November 18, 2016).

### Data analysis for scale development

Exploratory factor analysis with promax rotation and Cronbach’s alpha was carried out to elucidate the proper item set for the DS using items in which instructors obtained the highest mean score among the four respondent groups, which were viewed as essential items for evaluating PIF.

After items for the DS were fixed, the total and factor scores of the DS in the four respondent groups were analyzed to confirm that the scales could differentiate between different groups at different developmental stages. Also, influential factors for the DS, such as age, gender, and experience in playing the role of physician were analyzed.

SPSS version 23. (IBM, New York, NY) was used for all data analyses and calculation of DS factor scores.

## Results

### Demographic characteristics of the respondents

First, data from 14 respondents (five 4th-year medical students, seven 6th-year medical students, two 2nd-year residents) who chose option 4 (neutral) as the response for 27 items or more (87%) or for 23 sequential items (74%) were excluded as invalid data. A total of 322 respondents (response rate 53.7%) (118 4th-year medical students (47.8%) and 120 6th-year medical students (51.5%) at Kagoshima University School of Medicine, 47 2nd-year residents (73.4%) at Kagoshima University Hospital, and 37 medical doctors (66.1%) at community hospitals and private clinics who served as instructors for medical students) returned the questionnaire with valid responses for analysis. The mean ages of 4th-year medical students, 6th-year medical students, residents, and instructors were 24.2, 25.4, 29.7, and 55.2 years, respectively (Table 1). The ratio of male to female respondents was 1.20 for 4th-year medical students, 1.13 for 6th-year medical students, 2.46 for residents, and 11.3 for instructors, while nine respondents did not provide gender information. The mean length of clinical experience among instructors was 29.3 years (standard deviation 6.2 years, range 15–40 years).

**Table 1:** Demographic data of the respondents analyzed in this study

Group	Number of respondents		*	Age (years)				
				Mean	SD	Minimum	Median	Maximum
4th-year medical students	Male	61	56	25.0	(4.85)	21	23	40
	Female	51	41	23.0	(1.49)	22	22	29
	Unknown	6						
	Total	118	97	24.2	(3.92)	21	23	40
6th-year medical students	Male	63	54	25.7	(2.77)	23	25	38
	Female	56	43	25.0	(2.19)	23	25	34
	Unknown	1						
	Total	120	97	25.4	(2.54)	23	25	38
2nd-year residents	Male	32	31	30.3	(4.56)	26	29	46
	Female	13	12	28.3	(2.90)	26	27	34
	Unknown	2						
	Total	47	43	29.7	(4.23)	26	28	46
Instructors	Male	34	34	55.6	(5.94)	45	57	68
	Female	3	3	50.7	(7.77)	42	53	57
	Total	37	37	55.2	(6.13)	42	57	68
Total	Male	190						
	Female	123						
	Unknown	9						
	Total	322						

A total of 336 persons responded to the questionnaire and 14 were excluded because of invalid responses

*Number of respondents who answered questions on both gender and age

SD: standard deviation

### DS development

Among the 31 items, instructors had the highest mean scores for 18 items. Combining these 18 items with items describing attributes of stage 4 and 5 and reversely coded items for stage 2, 28 items were used for the next step, and 15 items became possible DS items based on exploratory factor analysis and reliability analysis (Table 2).

**Table 2:** Items used for the developing scale, and mean item scores for each respondent group

			Item score							
Item		Direction of coding	4th-year medical students		6th-year medical students		2nd-year residents		Instructors	
			Mean	(SD)	Mean	(SD)	Mean	(SD)	Mean	(SD)
1	I cannot tolerate that colleagues who sympathize with my actions have a different mindset from me.	R	5.56	(1.244)	5.51	(1.270)	5.51	(1.502)	**5.65**	(1.086)
2	I find it difficult to suppress my desires and act rationally.	R	5.05	(1.437)	5.08	(1.406)	**5.45**	(1.442)	5.41	(1.322)
3	It is difficult for me to adjust and act according to the different values of each medical professional and the demands for physicians.	R	4.19	(1.377)	4.20	(1.268)	4.34	(1.550)	**5.00**	(1.202)
4	I have never thought about the reasons or principles behind the required code of conduct.	R	4.56	(1.251)	4.33	(1.238)	4.51	(1.249)	**4.86**	(1.316)
5	I am sometimes unable to do something I was not interested in despite understanding its necessity.	R	3.84	(1.313)	4.23	(1.454)	**4.43**	(1.678)	4.27	(1.347)
6	The way I behave in medical settings is not my true self.	R	4.31	(1.167)	4.44	(1.346)	4.68	(1.400)	**5.46**	(1.366)
7	I behave correctly as a physician on a daily basis.	F	4.25	(0.999)	4.24	(1.108)	5.00	(1.319)	**5.41**	(1.235)
8	I am aware of my position as a physician.	F	4.97	(1.330)	4.68	(1.361)	6.26	(1.113)	**6.65**	(0.633)
9	I have accepted the words of gratitude and the frustration and anger of patients as a personal evaluation of myself.	F	4.86	(1.080)	5.18	(1.092)	5.13	(0.992)	**5.57**	(0.835)
10	I consider long-term significance and concerns when I think about what I should do now.	F	5.29	(1.087)	5.18	(1.112)	5.28	(1.136)	**5.54**	(0.767)
11	I have used my own beliefs and ideals as a standard to evaluate my own actions as a physician.	F	4.58	(1.024)	4.62	(1.030)	4.43	(1.156)	**4.89**	(0.994)
12	If I were able to play a role in improving society and organizations, I would be satisfied even if I did not receive individual recognition.	F	4.23	(1.386)	4.04	(1.411)	4.28	(1.347)	**4.57**	(1.425)
13	I induce action in the people around me based on the principles I believe in to fulfill my role as a physician.	F	4.48	(1.027)	4.38	(1.117)	4.43	(1.078)	**5.11**	(0.843)
14	I take on various roles in accordance with the demands of society.	F	4.57	(1.199)	4.28	(1.159)	5.13	(0.947)	**5.49**	(0.901)
15	I feel that I need to change my current mindset and everyday behavior.	R	3.14	(1.267)	3.24	(1.341)	3.32	(1.321)	**4.19**	(1.578)
	N		118 (117)		120 (119)		47 (46)		37	

Direction of coding: F, forward; R: reverse. SD: standard deviation; N: number of respondents

Bold indicates the highest score among the respondent groups

The Kaiser-Meyer Olkin Measure of sampling adequacy for the 15 items was 0.738 and Barlett’s test of sphericity was statistically significant (Chi-square = 932.51, df = 105; *p* < 0.01), suggesting that there was appropriate common variance and these items were interrelated. Exploratory factor analysis of these items using the scores of 318 respondents indicated a five-factor structure. The initial eigenvalues for factors 1 to 5 were 3.29, 2.20, 1.33, 1.05, and 1.03, respectively. The percentages of variance for factors 1 to 5 were 21.9, 14.7, 8.9, 7.0, and 6.9%, respectively. All items had a component coefficient over 0.4, and the cumulative percentage of all five factors was 59.3%. Cronbach’s alpha of the 15 items was 0.72.

Promax rotated pattern matrix coefficients are shown in Table 3. The names of the five factors were as follows: factor 1: self-control as a professional, factor 2: awareness of being a medical doctor, factor 3: reflection as a medical doctor, factor 4: execution of social responsibility, and factor 5: external and internal self-harmonization. Each factor score was computed using factor loadings and respondents’ scores of all 15 items, and the mean of each factor was standardized to be 0 and the variance was set to 1. Factor scores for each respondent group are shown in Table 4.

**Table 3:** Pattern matrix components of the five factors of the developing scale after factor analysis using Promax rotation with Kaiser normalization

Items		Pattern Matrix Component					Communalities
No.	Direction	1	2	3	4	5	Extraction
1	R	**0.74**	−0.01	0.14	−0.17	− 0.19	0.56
2	R	**0.66**	0.32	−0.14	− 0.02	− 0.10	0.54
3	R	**0.62**	0.04	−0.17	0.36	0.07	0.57
4	R	**0.59**	−0.23	0.31	0.09	−0.03	0.47
5	R	**0.56**	−0.17	− 0.22	0.13	0.29	0.50
6	R	**0.49**	0.01	0.30	−0.24	**0.41**	0.64
7		−0.02	**0.88**	−0.01	0.03	−0.04	0.77
8		−0.01	**0.84**	0.11	−0.06	−0.01	0.73
9		0.13	−0.03	**0.70**	0.11	−0.24	0.54
10		0.00	0.17	**0.68**	−0.10	0.05	0.53
11		−0.13	−0.05	**0.52**	0.37	−0.03	0.48
12		0.10	−0.09	−0.01	**0.80**	−0.29	0.61
13		−0.21	0.06	0.29	**0.53**	0.28	0.63
14		0.15	0.37	0.05	**0.50**	0.07	0.59
15	R	−0.04	−0.03	−0.15	−0.13	**0.89**	0.74
Rotation sums of squared loadings		2.552	2.240	2.026	1.979	1.568	

R: Coding direction was reversed

Bold indicates a loading ≥0.4

**Table 4:** Factor scores of the developing scale for each respondent group

	4th-year medical students *N* = 115		6th-year medical students *N* = 119		2nd-year residents *N* = 47		Instructors *N* = 37	
	Mean	(SD)	Mean	(SD)	Mean	(SD)	Mean	(SD)
Factor 1	−0.11	(0.92)	−0.07	(0.93)	0.16	(1.23)	0.37	(1.06)
Factor 2	−0.17	(0.94)	−0.36	(0.91)	0.62	(0.90)	0.92	(0.64)
Factor 3	−0.07	(1.02)	−0.05	(0.95)	−0.10	(1.11)	0.52	(0.81)
Factor 4	−0.01	(1.05)	−0.19	(0.88)	0.06	(1.12)	0.54	(0.85)
Factor 5	−0.16	(0.84)	−0.12	(1.02)	0.04	(0.93)	0.81	(1.13)

N: number of respondents; SD: standard deviation

Each factor score was computed by SPSS ver. 23 using factor loadings and respondent’ scores of all 15 items, and each factor was standardized to have a mean of 0 and a variance of 1

### Comparison of DS scores by respondent group

**Fig. 1:**
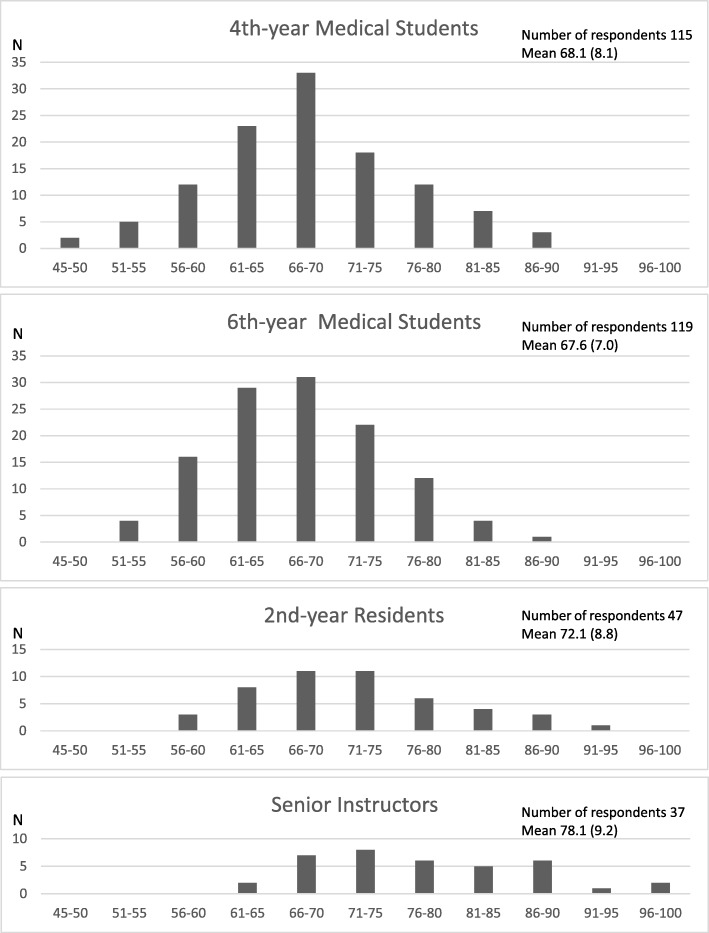
Score distribution of the developing scale (DS) in each respondent group. The DS consists of 15 items (Table 2) scored on a seven-point Likert scale that ranged from 1 (completely inapplicable) to 7 (greatly applicable), and 4 was neutral. The number of respondents (N) and total DS scores ranging from 45 to 100 for each respondent group (4th-year preclinical medical students, 6th-year medical students who finished 1.5 years of all clinical clerkship courses, 2nd-year residents, and medical doctors working in community hospitals or private clinics in Kagoshima Prefecture who engaged in undergraduate medical education as senior instructors [clinical experience mean 29.3, SD 6.2 years]) are shown. N: number of respondents; DS: developing scale; SD: standard deviation

Figure 1 shows the DS score distribution for each respondent group.

The mean DS score of the instructors was significantly higher than that of the residents (*p* < 0.01), the mean score of residents and instructors was significantly higher than that of students (p < 0.01), and the mean score of the instructors was significantly higher than that of all other respondents (p < 0.01). The number of respondents with a total score of 81 or higher was 10 (8.7%) for 4th-year medical students, five (4.2%) for 6th-year medical students, eight (17.0%) for residents, and 14 (37.8%) for instructors. The number of respondents with a total score of 91 or higher was none for 4th- and 6th-year medical students, one (2.1%) for residents, and three (8.1%) for instructors. The number of respondents with a total score of 60 or less was 19 (16.5%) for 4th-year medical students, 20 (16.8%) for 6th-year medical students, three (6.4%) for residents, and none for instructors.

Univariate analysis of variance of the DS score indicated that respondent group was a significant independent variable (*p* < 0.01), but gender was not. Univariate analysis of variance of the DS score in each respondent group indicated that gender and age were not significant independent variables in the four respondent groups, with the exception of age (*p* < 0.05) in 4th-year medical students.

### Experience in playing the role of a physician and DS and factor scores

Table 5 shows the mean scores of the four items related to role recognition (experience in playing the role of a physician and recognition as a physician by others, such as patients and families, senior physicians and supervisors, and nurses and other staff) for 6th-year medical students and residents.

**Table 5:** Scores of four items related to role recognition of 6th-year medical students (number of respondents was 118) and 2nd-year residents (number of respondents was 47), and correlation with developing scale score and factor scores

Items related to role recognition				Correlation						
Items		Respondents	Mean (SD)	DS		Factor score				
						Factor 1	Factor 2	Factor 3	Factor 4	Factor 5
a	***I*** have thought and acted as the lead physician of patients under my care.	6th-year medical students	4.6 (1.32)	r	**0.21** ^*^	0.031	**0.31** ^**^	0.17	0.12	0.01
				p	0.023	0.721	0.001	0.063	0.185	0.949
		2nd-year residents	5.6 (1.14)	r	0.20	−0.13	0.13	**0.33** ^*^	**0.48** ^**^	−0.21
				p	0.187	0.380	0.370	0.024	0.001	0.148
b	***The patients and families under my care*** have acknowledged and complied with me as their physician.	6th-year medical students	4.1 (1.49)	r	**0.19** ^*^	−0.03	**0.32** ^**^	0.16	**0.19** ^*^	0.00
				p	0.043	0.750	0.001	0.090	0.041	0.965
		2nd-year residents	4.9 (1.56)	r	0.25	−0.16	0.15	**0.41** ^**^	**0.56** ^**^	0.19
				p	0.092	0.270	0.317	0.005	0.000	0.190
c	***Senior physicians and supervisors*** have respected me as the lead physician of patients under my care.	6th-year medical students	4.4 (1.43)	r	0.17	−0.06	**0.29** ^**^	0.12	**0.19** ^*^	0.07
				p	0.061	0.495	0.001	0.210	0.045	0.474
		2nd-year residents	5.1 (1.24)	r	0.15	−0.19	0.23	**0.30** ^*^	**0.38** ^**^	−0.03
				p	0.329	0.193	0.113	0.038	0.009	0.853
d	***Nurses and other medical staff*** have complied with me as the lead physician of patients under my care.	6th-year medical students	3.6 (1.52)	r	0.13	−0.16	**0.35** ^**^	0.10	**0.26** ^**^	−0.01
				p	0.165	0.094	0.000	0.301	0.004	0.883
		2nd-year residents	4.9 (1.42)	r	0.19	−0.00	0.04	0.17	**0.38** ^**^	0.09
				p	0.198	0.992	0.790	0.242	0.008	0.535

r: Pearson Correlation coefficient; p: Significant value (2-tailed)

**(Bold): Correlation is significant at the 0.01 level; *(Bold): Correlation is significant at the 0.05 level

Correlation coefficients between those scores and the DS or the five factor scores indicated weak but significant correlations between 6th-year medical students’ scores and factors 2 and 4, and a strongly significant correlation between residents’ scores and factors 3 and 4.

## Discussion

This study attempted to develop an instrument to evaluate the degree of personal maturation and professional development in terms of socialization. The DS was designed to evaluate attributes of personally and professionally developed medical trainees and instructors received higher scores.

Exploratory factor analysis of the DS indicated a five-factor structure that explained 59% of the variance, and all five factors were included at the initial phase of item development as anticipated attributes of medical doctors.

The first factor of the DS, self-control as a professional, consisted of items describing uncontrolled emotional or irrational reactions in various situations and is typically seen in a person at Kegan’s stage 2 [17]; this factor was reversely scored for the DS. A previous qualitative study reported that medical students’ reflection on emotional experiences in the first clinical year related to rapid professional development [10], and managing emotions is one of the core components of emotional intelligence. Emotional intelligence is known as a key element of professionalism in health professionals [23–26], and emotional control is one of the dimensions of resilience that might be another anticipated attribute of health professionals [26, 27].

Scores of the second factor, awareness of being a medical doctor, were significantly higher for residents and instructors than medical students. This result is compatible with the fact that residents and instructors are socially approved licensed doctors in Japan, and indicates that formal qualification is not completely but closely related to actual behavior (item No. 7, 14) and recognition of professional identity (item No. 8).

The third factor, reflection as a medical doctor, indicated attitudes of self-evaluation based on external (patients’ perspectives, item No. 9) and internal values (item No. 11) and long-term significance (item No. 10) of one’s own behavioral standards. The fourth factor, execution of social responsibility, indicated that people obtain social perspectives. The third and fourth factors describe the attributes of autonomous reflective learners and social advocates that are essential for well-developed professionals.

The fifth factor, external and internal self-harmonization, consisted of items describing attributes seen at stage transition and were reversely scored for the DS. High scores in this factor indicated complete integrity of self and external values that characterize a person at Kegan’s stage 5 [17]. Professional trainees must face conflicted values and need to make adjustments throughout the process of PIF [28–30], and medical students and residents experience a mismatch between what they do and who they are [15, 31–34].

Holden et al. developed a six-domain framework of PIF for medical education [35]. The DS items and factors are related to Holden et al.’s five domains of personal characteristics, duties and responsibilities, habits, relationships, and perception and recognition. Since DS score increased as medical training advanced, the DS might serve as a measure of the essential elements of professional medical doctors.

Age, one of the indicators of personal maturation, was related to DS score in 4th-year medical students, but not in more advanced medical trainees and experienced physicians. The DS might evaluate personal development indicated by age before starting clinical training. After that, the DS may evaluate personal and professional development related to clinical experience.

The five factor scores indicated that attributes were not simultaneously developed. Interestingly, 6th-year medical students’ experiences in playing the role of a physician during clinical clerkship courses might induce awareness of being a medical doctor, while residents’ role experiences might facilitate reflection as a medical doctor and execution of social responsibility. Authentic and appropriate professional role experience is a well-known facilitator of PIF [8, 9, 16]. The present results suggest that the impact of experience in playing the role of a physician varies for medical trainees depending on different phases of PIF, and the DS and factor scores could be useful indicators of PIF.

### Limitations

Theoretical scale development using qualitative research data and group comparison were used for the DS validation. To clarify that the degree of personal maturation and professional development evaluated by the DS actually expresses the process of PIF, follow-up study of the same respondents is required.

In this research, the DS scores were not significantly different between 4th- and 6th-year medical students. It is well known that an individual’s role in society, or their work identity, facilitates professional identity construction [11, 36]. Sixth-year medical students might not have sufficient clinical experience to show apparent development from 4th-year medical students. In addition, 4th-year medical students responses might be incorrect or different from other respondents because their clinical experiences, such as observation and shadowing, do not require them to exercise clinical responsibility or manage conflict in clinical practice. Conformity of the target group with different experiences also needs to be analyzed to confirm the DS.

All items were written in Japanese and all respondents were located in Kagoshima, Japan. There is a possibility that some of the items may not be appropriate in other cultures. Further research with people in other locations is required.

## Conclusions

This is the first report to develop a scale that quantitatively evaluates young medical trainees’ PIF. The DS has a five-factor structure and evaluates self-control as a professional, awareness of being a medical doctor, reflection as a medical doctor, execution of social responsibility, and external and internal self-harmonization. Experience in playing the role of a physician might facilitate medical trainees’ PIF.

## Abbreviations

DS: developing scale
PIF: professional identity formation

## References

[1] Bebeau MJ, Kenny NP, Shelton WN (2006). Evidence based character development. Lost virtue professional character development in medical education.

[2] Cooke M, Irby DM, O’Brien BC (2010). Educating physicians: a call for reform of medical school and residency.

[3] Bleakley A, Bligh J, Bowne J (2011). Medical education for the future: identity, power and location.

[4] Cruess RL, Cruess SR, Boundreau JD, Snell L, Steinert Y (2014). Reframing medical education to support professional identity formation. Acad Med.

[5] Wilson I, Cowin LS, Johnson M, Young H (2013). Professional identity in medical students: pedagogical challenges to medical education. Teach Learn Med.

[6] Irby DM, Cooke M, O’Brien BC (2010). Call for reform of medical education by the Carnegie foundation for the advancement of teaching: 1910 and 2010. Acad Med.

[7] Javis-Selinger S, Pratt DD, Regehr G (2012). Competency is not enough: integrating identity formation into the medical education discourse. Acad Med.

[8] Lave J, Wenger E (1991). Situated learning: legitimate peripheral participation.

[9] Wenger E (1998). Communities of practice: learning meaning and identity.

[10] Pitkala KH, Mantyranta T (2003). Professional socialization revised: medical students’ own conceptions related to adoption of the future physician’s role—a qualitative study. Med Teacher.

[11] Pratt MG, Rockmann KW, Kaufmann JB (2006). Constructing professional identity: the role of work and identity learning cycles in the customization of identity among medical residents. Acad Manag J.

[12] Hafferty FW, Cruess RL, Cruess SR, Steinert Y (2009). Professionalism and the socialization of medical students. Teaching medical professionalism.

[13] Weaver R, Peters K, Koch J, Wilson I (2011). ‘Part of the team’: professional identity and social exclusivity in medical students. Med Educ.

[14] Frost HD, Regehr G (2013). “I am a doctor”: negotiating the discourses of standardization and diversity in professional identity construction. Acad Med.

[15] Cruess RL, Cruess SR, Boundreau JD, Snell L, Steinert Y (2015). A schematic representation of the professional identity formation and socialization of medical students and residents: a guide for medical educators. Acad Med.

[16] Sharpless J, Baldwin N, Cook R, Kofman A, Morley-Fletcher A, Slotkin R, Wald HS (2015). The becoming: students’ reflections on the process of professional identity formation in medical education. Acad Med.

[17] Kegan R (1982). The evolving self: problem and process in human development.

[18] Piaget J, Inhelder B (1969). The psychology of the child.

[19] Erikson EH (1982). The lifecycle completed.

[20] Kohlberg L (1984). The Physhology of moral development: moral stages and the lifecycle.

[21] Cruess RL, Cruess SR, Steinert Y (2016). Amending Miller’s pyramid to include professional identity formation. Acad Med.

[22] Forsythe GB (2005). Identity development in professional education. Acad Med.

[23] Feldman MD (2001). Becoming an emotionally intelligent physician. West J Med.

[24] Lewis NJ, Rees CE, Hudson N, Bleakley A (2005). Emotional intelligence in medical education: measuring the unmeasurable?. Adv Health Sci Educ.

[25] Tayler C, Farver C, Stoller JK (2011). Can emotional intelligence training serve as an alternative approach to teaching professionalism to residents?. Acad Med.

[26] Wald HS (2015). Professional identity (trans)formation in medical education: reflection, relationship, resilience. Acad Med.

[27] Tempki P, Martins MA, Paro HBMS (2012). Teaching and learning resilience: a new agenda in medical education. Med Educ.

[28] Ibarra H (1999). Provisional selves: experimenting with image and identity in professional adaptation. Admin Sci Quart.

[29] Bejaard D, Meijer PC, Verloop N (2004). Reconsidering research on teachers’ professional identity. Teach Teacher Educ.

[30] Ashforth BK, Sluss DM, Saks AM (2007). Socialization tactics, proactive behavior, and newcomer learning: integrating socialization models. J Voc Behavior.

[31] Ginsburg S, Regehr G, Hatala R, McNaughton N, Frohna A, Hodges B, Lingard L, Stern D (2000). Context, conflict, and resolution: a new conceptual framework for evaluating professionalism. Acad Med.

[32] White CB, Kumagai AK, Ross PT, Fantone JC (2009). A qualitative exploration of how the conflict between the formal and informal curriculum influences student values and behaviors. Acad Med.

[33] MacLeod A (2011). Caring, competence and professional identities in medical education. Adv in Health Sci Educ.

[34] Rees CE, Monrouxe LV, McDonald LA (2013). Narrative, emotion and action: analysing ‘most memorable’ professionalism dilemmas. Med Educ.

[35] Holden MD, Buck E, Luk J, Ambriz F, Boisaubin EV, Clark MA, Mihalic AP, Sadler JZ, Sapire KJ, Spike JP, Vince A, Dalrymple JL (2015). Professional identity formation: creating a longitudinal framework through TIME (transformation in medical education). Acad Med.

[36] Monrouxe LV, Rees CE, Hu W (2011). Difference in medical students’ explicit discourses of professionalism: acting, representing, becoming. Med Educ.

